# Association of *MTR* gene polymorphisms with the occurrence of non-syndromic congenital heart disease: a case–control study

**DOI:** 10.1038/s41598-023-36330-x

**Published:** 2023-06-09

**Authors:** Yiping Liu, Taowei Zhong, Xinli Song, Senmao Zhang, Mengting Sun, Jianhui Wei, Jing Shu, Tubao Yang, Tingting Wang, Jiabi Qin

**Affiliations:** 1 Department of Epidemiology and Health Statistics, Xiangya School of Public Health, Central South University, 110 Xiangya Road, Changsha, 410078 Hunan China; 2 Hunan Provincial Key Laboratory of Clinical Epidemiology, Changsha, Hunan China; 3 NHC Key Laboratory of Birth Defect for Research and Prevention, Hunan Provincial Maternal and Child Health Care Hospital, 53 Xiangchun Road, Changsha, 410028 Hunan China

**Keywords:** Genetics, Cardiology

## Abstract

To exhaustively explore the association of infant genetic polymorphisms of methionine synthase (*MTR*) gene with the risk of non-syndromic congenital heart disease (CHD). A hospital-based case–control study involving 620 CHD cases and 620 health controls was conducted from November 2017 to March 2020. Eighteen SNPs were detected and analyzed. Our date suggested that the genetic polymorphisms of *MTR* gene at rs1805087 (GG vs. AA: aOR = 6.85, 95% CI 2.94–15.96; the dominant model: aOR = 1.77, 95% CI 1.35–2.32; the recessive model: aOR = 6.26, 95% CI 2.69–14.54; the addictive model: aOR = 1.81, 95% CI 1.44–2.29) and rs2275565 (GT vs. GG: aOR = 1.52, 95% CI 1.15–1.20; TT vs. GG: aOR = 4.93, 95% CI 1.93–12.58; the dominant model: aOR = 1.66, 95% CI 1.27–2.17; the recessive model: aOR = 4.41, 95% CI 1.73–11.22; the addictive model: aOR = 1.68, 95% CI 1.32–2.13) were significantly associated with the higher risk of CHD. And three haplotypes of G-A-T (involving rs4659724, rs95516 and rs4077829; OR = 5.48, 95% CI 2.58–11.66), G-C-A-T-T-G (involving rs2275565, rs1266164, rs2229276, rs4659743, rs3820571 and rs1050993; OR = 0.78, 95% CI 0.63–0.97) and T-C-A-T-T-G (involving rs2275565, rs1266164, rs2229276, rs4659743, rs3820571 and rs1050993; OR = 1.60, 95% CI 1.26–2.04) were observed to be significantly associated with risk of CHD. Our study found that genetic polymorphisms of *MTR* gene at rs1805087 and rs2275565 were significantly associated with higher risk of CHD. Additionally, our study revealed a significant association of three haplotypes with risk of CHD. However, the limitations in this study should be carefully taken into account. In the future, more specific studies in different ethnic populations are required to refine and confirm our findings.

**Trial registration:** Registration number: ChiCTR1800016635; Date of first registration: 14/06/2018.

## Introduction

Congenital heart disease (CHD) refers to a gross structural abnormality in heart or intrathoracic great vessels occurring in the embryonic period^1^. It is the most common human birth defect and the primary non-infectious cause of infant mortality^2,3^. The incidence of CHD worldwide is estimated to be approximately 8.22 per 1000 live births and rising^4,5^. Over the past few decades, rapid advances in surgical treatments and interventional therapies decreased the mortality of CHD^6^. Nevertheless, its associated complications (such as arrhythmia, heart failure and sudden cardiac death) and neurodevelopmental disorders may still occur after effective correction of cardiac abnormalities^7–9^.

CHD is a complicated pathological progress which can be influenced by environmental factors or genetic factors, or a combination of two factors^10,11^. Researches show maternal extrinsic and intrinsic factors may increase CHD risk, such as folate deficiency, diabetes, obesity and so on^12,13^. However, not all pregnant women who are exposed to risk factors will bear CHD infants, which demonstrates that individual’s susceptibility to CHD may vary depending on genetic factors. Mounting evidence supports that genetic risk factors play a crucial role in the etiology of CHD^14^. Further exploration of the hereditary etiology of CHD will likely be an essential step to improve treatments for patients with CHD.

Methionine synthase (*MTR*) gene, encoding methionine synthase, map to chromosomes 1q43. MTR, the major regulatory enzyme involved in folate/homocysteine (Hcy) metabolic pathway, which can transfer the methyl group from 5-methyltetrahydrofolic acid to Hcy and produce methionine and tetrahydrofolic acid by means of remethylation^15^. It is the only way to decrease the concentration of plasma Hcy in the early stage of embryo development. When *MTR* is insufficient or inactive, homocysteine will accumulate in the body resulting in hyperhomocysteinemia (HHcy). Published literature have found that HHcy might be a risk factor of birth defects including CHD^16,17^. Hence a presumption was raised that the genetic variants of *MTR* gene may alter the susceptibility to CHD by affecting the folate/Hcy metabolism.

Presently, some studies focused on 1–2 loci of the *MTR* gene when assessing the association of *MTR* genetic polymorphisms with the risk of CHD. The present study is the first to comprehensively evaluate the 18 single nucleotide polymorphisms (SNPs) of *MTR* gene and the risk of CHD in Han Chinese populations.

## Materials and methods

### Recruitments of study participants

From November, 2017 to March, 2020, all consenting children in the study were recruited from the Hunan Children's Hospital, Hunan Province, China. Children recruited from Department of Cardiothoracic Surgery with CHD were selected as the case group. The diagnosis and classification of the CHD were confirmed by echocardiography and/or surgery. During the same period. children recruited from Department of Child Healthcare without any congenital malformations after medical examination were selected as the control group.

Ethical approval was given by the Ethics Committee of Xiangya School of Public Health, Central South University (No. XYGW-2018–36). Additionally, it had been registered in Chinese Clinical Trial Registry Center with registration number ChiCTR1800016635 (date of first registration: 14/06/2018) and is available at http://www.chictr.org.cn/listbycreater.aspx. Information and biological samples were collected from participants after obtaining written informed consent from their parents.

### Inclusion and exclusion criteria

In the present study, CHD was the outcome of interest which included ventricular septal defect (VSD), atrial septal defect (ASD), atrioventricular septal defect (AVSD), patent ductus arteriosus (PDA), aorto-pulmonary window (APW), tetralogy of Fallot (TOF) and complete transposition of great arteries (TGA). Of note, this study concerned on non-syndromic CHD. Patients with other organ malformations or known chromosomal abnormalities were excluded. We required that all case and controls were Han Chinese descent to reduce residual confounding factors from genetic and cultural differences owing to different ethnics. To minimize potential recall bias of exposure by mothers during the pre-pregnancy to the early stage of this pregnancy, we only included the study subjects who were less than 1 year old. Additionally, the case and controls should meet the following inclusion criteria: 1) child whose mother was spontaneous pregnancy; 2) singleton pregnancy; 3) completed the questionnaire and provided the blood sample; 4) child whose mother was unreported history of depression or other psychiatric disorders.

### Information collection

Considering the influence of potential confounding factors in the later analysis, the trained investigators used self-designed questionnaire to collect the corresponding information through one-to-one interview. The covariates for mothers were pre-selected based on literature review as followings: age at this pregnancy (< *35 or* ≥ *35*), residence (*rural or urban*), body mass index (BMI) before pregnancy(< *18.5, 18.5–24.9, 24.9–29.9 or* ≥ *30.0*), history of gestational diabetes mellitus (*yes or no*), history of gestational hypertension(*yes or no*), history of consanguineous marriage(*yes or no*), family history of congenital malformations(*yes or no*), cold before pregnancy(*yes or no*), smoking before pregnancy(*yes or no*), alcohol drinking before pregnancy(*yes or no*), folic acid consumption before or during pregnancy(*yes or no*). We defined folic acid consumption as any use of folic acid in 3 months before pregnancy and/or during the first-trimester pregnancy. To reduce recall bias to some extent, the exposure information was further confirmed by consulting their Maternal and Child Health Manual and medical records.

When mothers completed the questionnaires mentioned above, three milliliters of peripheral venous blood were provided from their children and collected in EDTA treated (ethylenediamine tetra acetic acid) anticoagulant tubes. And then blood samples immediately centrifuged into plasma and blood cells. Blood cells were separated and stored at − 80 °C until genotyping analysis. Genomic DNA was isolated from peripheral blood cells using the QIAamp DNA Mini Kit (Qiagen, Valencia, CA), and dissolved in sterile TBE buffer based on the manufacturer’s protocol.

### SNP selection and genotyping

*MTR* gene was the candidate gene for this study. We selected the corresponding candidate loci of the *MTR* gene based on a previously published study^18^. Briefly, SNP tags were selected using the SNPBrowser™ (version 3.0). This program was provided by AppliedBiosystems Inc which allowed selection of SNP markers from the HapMap database (http://www.hapmap.org/). For each target gene, tagging SNPs were selected based on the pairwise r^2^ ≥ 0.8. We excluded these SNPs with minor allele frequencies lower than 10% in Asians. Finally, these genetic loci (rs1266164, rs3768139, rs6676866, rs4077829, rs955516, rs1050993, rs2229276, rs4659743, rs12060570, rs1806505, rs3768142, rs4659724, rs6668344, rs1805087, rs2275565, rs3754255, rs10925252, rs3820571) of *MTR* gene, were considered as candidate loci in our study.

We used the matrix-assisted laser desorption and ionization time-of-flight mass spectrometry Mass Array system (Agena iPLEXassay, San Diego, CA, USA) to genotype the polymorphisms of *MTR* gene. The laboratory technician, who performed the genotyping, retyped and double-checked each sample, and recorded the genotype data, was blinded to whether the samples were from cases or controls. We set the minimum call rate of SNP genotyping at the level of 50% to ensure data integrity of the participant’s genotypes that had been called.

### Statistical analysis

Categorical variables were described as frequencies and percentages. The Chi-square Test was used to compare the differences in qualitative demographic features between the case group and the control group. Hardy–Weinberg equilibrium (HWE) was tested using the goodness-of-fit Chi-square Test in the controls. In the present study, we comprehensively analyzed the association of genotype and three genetic models (i.e., *dominant model, recessive model and additive model*) for every SNP with the risk of CHD. Odds ratio (OR) and 95% confidence intervals (CIs) were calculated using logistic regression analysis to evaluate the associations between *MTR* gene polymorphism and CHD risk. Adjusted OR (aOR) was calculated by multivariable logistic regression to adjust statistically significant variables of maternal characteristics between case and control groups, which aims to further evaluate independent association of SNPs of the *MTR* gene on the susceptibility of CHD. Besides, to get a more precise *P* value, the false discovery rate (FDR_*P*) was applied to multiple test corrections. Linkage disequilibrium test was used to evaluate whether there was a strong association between the two SNPs. Associations between haplotype and the risk of CHD were estimated by haplotype analysis. Linkage disequilibrium test and haplotype analysis was conducted using Haploview 4.2 software. Other statistical analyses were conducted using R software (version 3.5.0). All tests were two-tailed, with *P* < 0.05 set as the statistically significant difference.

### Ethics approval and consent to participate

This study was performed in line with the principles of the Declaration of Helsinki. Approval was granted by the Ethics Committee of the Xiangya School of Public Health of Central South University (No. XYGW-2018-36), and written informed consent was obtained from all mothers. The protocol of this study was registered at the Chinese Clinical Trial Registry with registration number ChiCTR1800016635 and is available at http://www.chictr.org.cn/listbycreater.aspx.

## Results

### Sociodemographic characteristics

Based on inclusion criteria, a total of 620 children with CHD were recruited into the case group, 620 healthy children into the control group. Among 620 CHD cases, 139 were diagnosed with ASD, 448 with VSD, 25 with AVSD, 168 with PDA, 32 with TOF, 7 with APW, and 3 with TGA. Considering that some cases have been diagnosed with multiple subtypes of CHD, the sum of the various subtypes was not equal to 620. Comparison of maternal characteristics in cases and controls were summarized in Table 1. Significant differences were found in case and control groups for BMI before pregnancy, history of gestational diabetes mellitus, history of gestational hypertension, history of consanguineous marriage, family history of congenital malformations, cold before pregnancy, smoking before pregnancy, alcohol drinking before pregnancy, folic acid consumption before or during pregnancy. These potential confounding factors were adjusted when estimating the association between *MTR* gene polymorphisms with the risk of CHD.

**Table 1:** Comparison of maternal characteristics in cases and controls ^a^.

Variables	Case (n = 620)	Control (n = 620)	Univariate analysis^b^
Age at this pregnancy (Years)	–	–	χ^2^ = 0.993; *P* = 0.319
< 35	546(88.1%)	557(89.8%)	–
≥ 35	74(11.9%)	63(10.2%)	
Residence	–	–	χ^2^ = 36.153; *P* < 0.001
Rural	444(71.6%)	342(55.2%)	–
Urban	176(28.4%)	278(44.8%)	
BMI before pregnancy	–	–	χ^2^ = 18.353; *P* < 0.001
< 18.5	112(18.1%)	156(25.2%)	
18.5–24.9	425(68.5%)	382(61.6%)	
24.9–29.9	62(10.0%)	43(6.9%)	
≥ 30.0	21(3.4%)	39(6.3%)	
History of gestational diabetes mellitus(yes)	63(10.2%)	17(2.7%)	χ^2^ = 28.274; *P* < 0.001
History of gestational hypertension(yes)	43(6.9%)	9(1.5%)	χ^2^ = 23.204;* P* < 0.001
History of consanguineous marriage(yes)	21(3.4%)	3(0.5%)	χ^2^ = 13.766; *P* < 0.001
Family history of congenital malformations(yes)	36(5.8%)	5(0.8%)	χ^2^ = 24.241; *P* < 0.001
Cold before pregnancy(yes)	121(19.5)	72(11.6%)	χ^2^ = 14.734; *P* < 0.001
Smoking before pregnancy(yes)	36(5.8)	13(2.1%)	χ^2^ = 11.240; *P* = 0.001
Alcohol drinking before pregnancy(yes)	81(13.1%)	43(6.9%)	χ^2^ = 12.939; *P* < 0.001
Folic acid consumption before or during pregnancy(yes)	526(84.8%)	577(93.1%)	χ^2^ = 21.344; *P* < 0.001

^a^ Data presented as number (percentage) unless otherwise indicated;

^b^ *P* < 0.05 was considered to indicate a statistically significant difference.

### Genetic variants of *MTR* gene and risk of CHD

*MTR* genotype frequencies in infant and the results of HWE test were shown in Table 2. The genotype frequencies of the 18 SNPs of *MTR* gene in the control group all conformed to HWE (all *P* values > 0.05).

**Table 2:** Genotype frequencies of *MTR* gene and HWE test of the control group^a^.

Genetic loci	Chromosome	Major allele	Minor allele	MAF	Group	Genotype frequencies			*P*^b^
						AA	AB	BB^c^	
rs955516	1:236,980,504	T	A	0.500	Control	196 (31.6%)	314(50.6%)	110(17.8%)	0.414
					Case	206(33.2%)	298(48.1%)	116(18.7%)	
rs1266164	1:237,050,951	C	T	0.167	Control	427(68.9%)	168(27.1%)	25(4.0%)	0.106
					Case	411(66.3%)	185(29.8%)	24(3.9%)	
rs3768139	1:237,027,668	C	G	0.183	Control	424(68.4%)	171(27.6%)	25(4.0%)	0.146
					Case	411(66.3%)	185(29.8%)	24(3.9%)	
rs6676866	1:237,064,626	G	T	0.358	Control	221(35.6%)	285(46.0%)	114(18.4%)	0.192
					Case	190(30.6%)	314(50.6%)	116(18.8%)	
rs1050993	1:237,062,305	G	A	0.189	Control	418(67.5%)	177(28.5%)	25(4.0%)	0.257
					Case	408(65.8%)	185(29.8%)	27(4.4%)	
rs2229276	1:237,054,569	A	G	0.484	Control	175(28.2%)	307(49.5%)	138(22.3%)	0.879
					Case	182(29.4%)	302(48.7%)	136(21.9%)	
rs4659743	1:237,059,387	T	A	0.173	Control	420(67.8%)	175(28.2%)	25(4.0%)	0.215
					Case	406(65.5%)	183(29.5%)	31(5.0%)	
rs1806505	1:236,996,575	C	T	0.490	Control	190(30.6%)	320(51.6%)	110(17.8%)	0.216
					Case	219(35.3%)	293(47.3%)	108(17.4%)	
rs3768142	1:237,028,564	T	C	0.294	Control	260(41.9%)	269(43.4%)	91(14.7%)	0.119
					Case	248(40.0%)	273(44.0%)	99(16.0%)	
rs4659724	1:236,974,124	G	A	0.437	Control	192(31.0%)	316(51.0%)	112(18.0%)	0.362
					Case	239(38.5%)	279(45.0%)	102(16.5%)	
rs6668344	1:237,001,326	C	T	0.484	Control	174(28.1%)	310(50.0%)	136(21.9%)	0.925
					Case	188(30.3%)	305(49.2%)	127(20.5%)	
rs1805087	1:237,048,500	A	G	0.181	Control	486(78.4%)	127(20.5%)	7(1.1%)	0.685
					Case	408(65.8%)	175(28.2%)	37(6.0%)	
rs4077829	1:236,987,790	G	T	0.440	Control	194(31.3%)	316(51.0%)	110(17.7%)	0.339
					Case	201(32.4%)	308(49.7%)	111(17.9%)	
rs2275565	1:237,048,676	G	T	0.213	Control	475(76.6%)	139(22.4%)	6(1.0%)	0.230
					Case	412(66.5%)	181(29.1%)	27(4.4%)	
rs3754255	1:237,009,857	T	C	0.440	Control	172(27.7%)	301(48.6%)	147(23.7%)	0.494
					Case	179(28.9%)	286(46.1%)	155(25.0%)	
rs3820571	1:237,060,433	T	G	0.189	Control	417(67.3%)	175(28.2%)	28(4.5%)	0.086
					Case	416(67.1%)	180(29.0%)	24(3.9%)	
rs10925252	1:237,022,362	C	T	0.490	Control	145(23.4%)	298(48.1%)	177(28.5%)	0.368
					Case	156(25.2%)	276(44.5%)	188(30.3%)	
rs12060570	1:236,989,069	G	C	0.500	Control	193(31.1%)	317(51.1%)	110(17.8%)	0.304
					Case	224(36.1%)	285(46.0%)	111(17.9%)	

MAF minimum allele frequency.

^a^ Data presented as number (percentage) unless otherwise indicated;

^b^ *P* < 0.05 was considered to indicate a statistically significant difference;

^c^ AA, Homezygous with minor allele; AB, Heterozygous; BB, Homezygous with major allele.

Associations between each SNP of *MTR* gene and the risk of CHD based on univariate and multivariate logistic regression analysis were summarized in Table 3. After adjustment, the genetic polymorphism of *MTR* gene at rs1805087 was significantly associated with the higher risk of CHD (GG vs. AA: aOR = 6.85, 95% CI 2.94–15.96; the dominant model: aOR = 1.77, 95% CI 1.35–2.32; the recessive model: aOR = 6.26, 95% CI 2.69–14.54; the addictive model: aOR = 1.81, 95% CI 1.44–2.29). Additionally, the genetic polymorphism of *MTR* gene at rs2275565 was significantly associated with the higher risk of CHD (GT vs. GG: aOR = 1.52, 95% CI 1.15–1.20; TT vs.GG: aOR = 4.93, 95% CI 1.93–12.58; the dominant model: aOR = 1.66, 95% CI 1.27–2.17; the recessive model: aOR = 4.41, 95% CI 1.73–11.22; the addictive model: aOR = 1.68, 95% CI 1.32–2.13). However, statistically significant associations between the risk of CHD and genetic polymorphisms at other loci of *MTR* gene were not observed.

**Table 3:** Genetic variants of *MTR* gene and risk of CHD.

Genotype	Univariate analysis			Multivariate analysis		
	*P*	OR (95% CI)	FDR_*P*	*P*	aOR (95% CI) ^b^	FDR_*P*^a^
rs955516						
TT	1	1	1	1	1	1
TA	0.427	0.90(0.70–1.16)	0.834	0.384	0.89(0.67–1.16)	0.864
AA	0.984	1.00(0.72–1.39)	0.993	0.586	1.10(0.78–1.56)	0.884
Dominant model^c^	0.544	0.93(0.73–1.18)	0.703	0.641	0.94(0.73–1.22)	0.869
Recessive model^d^	0.659	1.07(0.80–1.42)	0.941	0.276	1.19(0.87–1.62)	0.954
Additive mode^e^	0.871	0.99(0.84–1.16)	0.990	0.767	1.03(0.86–1.22)	0.860
rs1266164						
CC	1	1	1	1	1	1
CT	0.289	1.14(0.89–1.47)	0.834	0.285	1.16(0.89–1.51)	0.864
TT	0.993	0.10(0.56–1.78)	0.993	0.684	0.88(0.47–1.65)	0.884
Dominant model^c^	0.332	1.13(0.89–1.43)	0.703	0.387	1.12(0.87–1.45)	0.697
Recessive model^d^	0.884	0.96(0.54–1.70)	0.941	0.583	0.84(0.45–1.57)	0.954
Additive mode^e^	0.445	1.08(0.86–1.32)	0.801	0.590	1.06(0.86–1.32)	0.860
rs3768139						
CC	1	1	1	1	1	1
GC	0.386	1.12(0.87–1.43)	0.834	0.373	1.13(0.87–1.47)	0.864
GG	0.974	0.99(0.56–1.76)	0.993	0.667	0.87(0.47–1.63)	0.884
Dominant model^c^	0.431	1.10(0.87–1.40)	0.703	0.487	1.10(0.85–1.41)	0.797
Recessive model^d^	0.884	0.96(0.54–1.70)	0.941	0.583	0.84(0.45–1.57)	0.954
Additive mode^e^	0.541	1.06(0.87–1.30)	0.819	0.692	1.05(0.84–1.30)	0.860
rs6676866						
GG	1	1	1	1	1	1
GT	0.053	1.28(0.10–1.65)	0.273	0.083	1.27(0.97–1.67)	0.383
TT	0.307	1.18(0.86–1.64)	0.834	0.806	1.05(0.74–1.48)	0.884
Dominant model^c^	0.062	1.25(0.99–1.59)	0.227	0.153	1.20(0.93–1.55)	0.424
Recessive model^d^	0.884	1.02(0.77–1.36)	0.941	0.542	0.91(0.67–1.24)	0.954
Additive mode^e^	0.183	1.11(0.95–1.31)	0.672	0.532	1.06(0.89–1.25)	0.860
rs1050993						
GG	1	1	1	1	1	1
GA	0.587	1.07(0.84–1.37)	0.879	0.546	1.09(0.83–1.42)	0.884
AA	0.724	1.11(0.63–1.94)	0.916	0.901	1.04(0.57–1.91)	0.954
Dominant model^c^	0.547	1.08(0.85–1.36)	0.703	0.556	1.08(0.84–1.39)	0.834
Recessive model^d^	0.777	1.08(0.62–1.89)	0.941	0.965	1.01(0.56–1.85)	0.970
Additive mode^e^	0.546	1.06(0.87–1.30)	0.819	0.611	1.06(0.85–1.31)	0.860
rs2229276						
AA	1	1	1	1	1	1
AG	0.676	0.95(0.73–1.23)	0.901	0.976	1.00(0.76–1.34)	0.976
GG	0.738	0.95(0.69–1.30)	0.916	0.628	1.09(0.77–1.53)	0.884
Dominant model^c^	0.661	0.95(0.74–1.21)	0.709	0.830	1.03(0.79–1.35)	0.934
Recessive model^d^	0.891	0.98(0.75–1.28)	0.941	0.578	1.08(0.82–1.44)	0.954
Additive mode^e^	0.719	0.97(0.83–1.14)	0.990	0.642	1.04(0.88–1.23)	0.860
rs4659743						
TT	1	1	1	1	1	1
TA	0.535	1.08(0.84–1.39)	0.875	0.480	1.10(0.84–1.44)	0.884
AA	0.370	1.28(0.74–2.21)	0.834	0.461	1.25(0.70–2.23)	0.884
Dominant model^c^	0.399	1.11(0.87–1.40)	0.703	0.384	1.12(0.87–1.44)	0.697
Recessive model^d^	0.413	1.25(0.73–2.15)	0.941	0.519	1.21(0.68–2.15)	0.954
Additive mode^e^	0.320	1.10(0.91–1.34)	0.799	0.340	1.11(0.90–1.37)	0.860
rs1806505						
CC	1	1	1	1	1	1
CT	0.072	0.79(0.62–1.02)	0.288	0.116	0.80(0.61–1.06)	0.464
TT	0.339	0.85(0.61–1.18)	0.834	0.646	0.92(0.65–1.31)	0.884
Dominant model^c^	0.080	0.81(0.64–1.03)	0.240	0.165	0.83(0.65–1.08)	0.424
Recessive model^d^	0.881	0.98(0.73–1.31)	0.941	0.751	1.05(0.77–1.44)	0.970
Additive mode^e^	0.205	0.90(0.77–1.06)	0.672	0.448	0.94(0.79–1.11)	0.860
rs3768142						
TT	1	1	1	1	1	1
GT	0.616	1.06(0.84–1.36)	0.879	0.575	1.08(0.83–1.40)	0.884
GG	0.440	1.14(0.82–1.59)	0.834	0.939	0.99(0.69–1.42)	0.966
Dominant model^c^	0.488	1.08(0.86–1.36)	0.703	0.676	1.05(0.83–1.34)	0.869
Recessive model^d^	0.528	1.11(0.81–1.51)	0.941	0.761	0.95(0.68–1.33)	0.970
Additive mode^e^	0.421	1.07(0.91–1.25)	0.801	0.889	1.01(0.85–1.20)	0.889
rs4659724						
GG	1	1	1	1	1	1
GA	**0.007**	**0.71(0.55–0.91)**	**0.050**	**0.017**	**0.72(0.55–0.942)**	0.122
AA	0.062	0.73(0.53–1.02)	0.279	0.235	0.81(0.57–1.15)	0.846
Dominant model^c^	**0.005**	**0.72(0.57–0.90)**	**0.030**	**0.022**	**0.74(0.58–0.96)**	0.132
Recessive model^d^	0.452	0.89(0.67–1.20)	0.941	0.906	0.98(0.72–1.34)	0.970
Additive mode^e^	**0.021**	**0.83(0.71–0.97)**	0.126	0.106	0.87(0.73–1.03)	0.636
rs6668344						
CC	1	1	1	1	1	1
CT	0.480	0.91(0.70–1.18)	0.857	0.789	0.96(0.73–1.28)	0.884
TT	0.368	0.86(0.63–1.19)	0.834	0.810	0.96(0.68–1.35)	0.884
Dominant model^c^	0.382	0.90(0.70–1.15)	0.703	0.771	0.96(0.74–1.26)	0.925
Recessive model^d^	0.532	0.92(0.70–1.20)	0.941	0.908	0.98(0.74–1.32)	0.970
Additive mode^e^	0.355	0.93(0.79–1.09)	0.799	0.799	0.98(0.83–1.16)	0.860
rs1805087						
AA	1	1	1	1	1	1
AG	** < 0.001**	**1.64(1.26–2.14)**	** < 0.001**	**0.006**	**1.49(1.12–1.98)**	0.054
GG	** < 0.001**	**6.30(2.78–14.27)**	** < 0.001**	** < 0.001**	**6.85(2.94–15.96)**	** < 0.001**
Dominant model^c^	** < 0.001**	**1.89(1.46–2.43)**	** < 0.001**	** < 0.001**	**1.77(1.35–2.32)**	** < 0.001**
Recessive model^d^	** < 0.001**	**5.56(2.46–12.57)**	** < 0.001**	** < 0.001**	**6.26(2.69–14.54)**	** < 0.001**
Additive mode^e^	** < 0.001**	**1.88(1.51–2.34)**	** < 0.001**	** < 0.001**	**1.81(1.44–2.29)**	** < 0.001**
rs4077829						
GG	1	1	1	1	1	1
GT	0.635	0.94(0.73–1.21)	0.879	0.771	0.96(0.73–1.26)	0.884
TT	0.875	0.97(0.70–1.35)	0.993	0.733	1.06(0.75–1.52)	0.884
Dominant model^c^	0.670	0.95(0.75–1.21)	0.709	0.918	0.99(0.76–1.28)	0.964
Recessive model^d^	0.941	1.01(0.76–1.35)	0.941	0.583	1.09(0.80–1.49)	0.954
Additive mode^e^	0.805	0.98(0.83–1.15)	0.990	0.812	1.02(0.86–1.22)	0.860
rs2275565						
GG	1	1	1	1	1	1
GT	**0.002**	**1.50(1.16–1.94)**	**0.018**	**0.003**	**1.52(1.15–1.20)**	**0.036**
TT	** < 0.001**	**5.19(2.12–12.69)**	** < 0.001**	**0.001**	**4.93(1.93–12.58)**	**0.018**
Dominant model^c^	** < 0.001**	**1.65(1.29–2.12)**	** < 0.001**	** < 0.001**	**1.66(1.27–2.17)**	** < 0.001**
Recessive model^d^	**0.001**	**4.66(1.91–11.37)**	** < 0.001**	**0.002**	**4.41(1.73–11.22)**	**0.018**
Additive mode^e^	** < 0.001**	**1.68(1.34–2.10)**	** < 0.001**	** < 0.001**	**1.68(1.32–2.13)**	** < 0.001**
rs3754255						
TT	1	1	1	1	1	1
TC	0.500	0.91(0.70–1.19)	0.857	0.302	0.86(0.65–1.14)	0.864
CC	0.934	1.01(0.75–1.38)	0.993	0.565	0.91(0.65–1.27)	0.884
Dominant model^c^	0.659	0.95 (0.74–1.21)	0.709	0.327	0.88(0.67–1.14)	0.697
Recessive model^d^	0.597	1.07(0.84–1.39)	0.941	0.970	0.10(0.75–1.32)	0.970
Additive mode^e^	0.969	1.00(0.86–1.17)	1.000	0.523	0.95(0.80–1.12)	0.860
rs3820571						
TT	1	1	1	1	1	1
TG	0.809	1.03(0.80–1.32)	0.971	0.726	1.05(0.80–1.37)	0.884
GG	0.597	0.86(0.49–1.51)	0.879	0.357	0.75(0.41–1.38)	0.864
Dominant model^c^	0.952	1.01(0.80–1.28)	0.952	0.964	1.01(0.78–1.30)	0.964
Recessive model^d^	0.571	0.85(0.49–1.49)	0.941	0.329	0.74(0.40–1.36)	0.954
Additive mode^e^	0.880	0.99(0.81–1.20)	0.990	0.757	0.97(0.78–1.20)	0.860
rs10925252						
CC	1	1	1	1	1	1
CT	0.293	0.86(0.65–1.14)	0.834	0.085	0.77(0.57–1.04)	0.383
TT	0.934	0.99(0.73–1.34)	0.993	0.357	0.86(0.62–1.19)	0.864
Dominant model^c^	0.466	0.91(0.70–1.18)	0.703	0.117	0.80 (0.61–1.06)	0.424
Recessive model^d^	0.493	1.09(0.85–1.39)	0.941	0.909	1.02(0.78–1.33)	0.970
Additive mode^e^	1.000	1.00(0.86–1.16)	1.000	0.390	0.93(0.79–1.10)	0.860
rs12060570						
GG	1	1	1	1	1	1
GC	**0.046**	**0.78(0.60–0.99)**	0.273	0.061	0.77(0.59–1.01)	0.366
CC	0.401	0.87(0.63–1.21)	0.834	0.775	0.95(0.67–1.35)	0.884
Dominant model^c^	0.063	0.80 (0.63–1.01)	0.227	0.121	0.82(0.63–1.06)	0.424
Recessive model^d^	0.941	1.01(0.76–1.35)	0.941	0.508	1.11(0.81–1.51)	0.954
Additive mode^e^	0.224	0.91(0.77–1.06)	0.672	0.499	0.94(0.79–1.12)	0.860

aOR = adjusted odds ratio; CI = confidence interval.

^a^ *P* < 0.05 was considered to indicate a statistically significant difference;

^b^ Adjusted for residence, BMI before pregnancy, history of gestational diabetes mellitus, history of gestational hypertension, history of consanguineous marriage, family history of congenital malformations, cold before pregnancy, smoking before pregnancy, alcohol drinking before pregnancy, and folate supplement before or during pregnancy.

^c^ Dominant model means homozygous variant + heterozygous variant versus homozygous wild-type.

^d^ Recessive model means homozygous variant versus heterozygous variant + homozygous wild-type.

^e^ Additive model means homozygous variant versus heterozygous variant versus homozygous wild-type.

Significant values are in [bold].

### Linkage disequilibrium test and haplotype analysis

**Figure 1:**
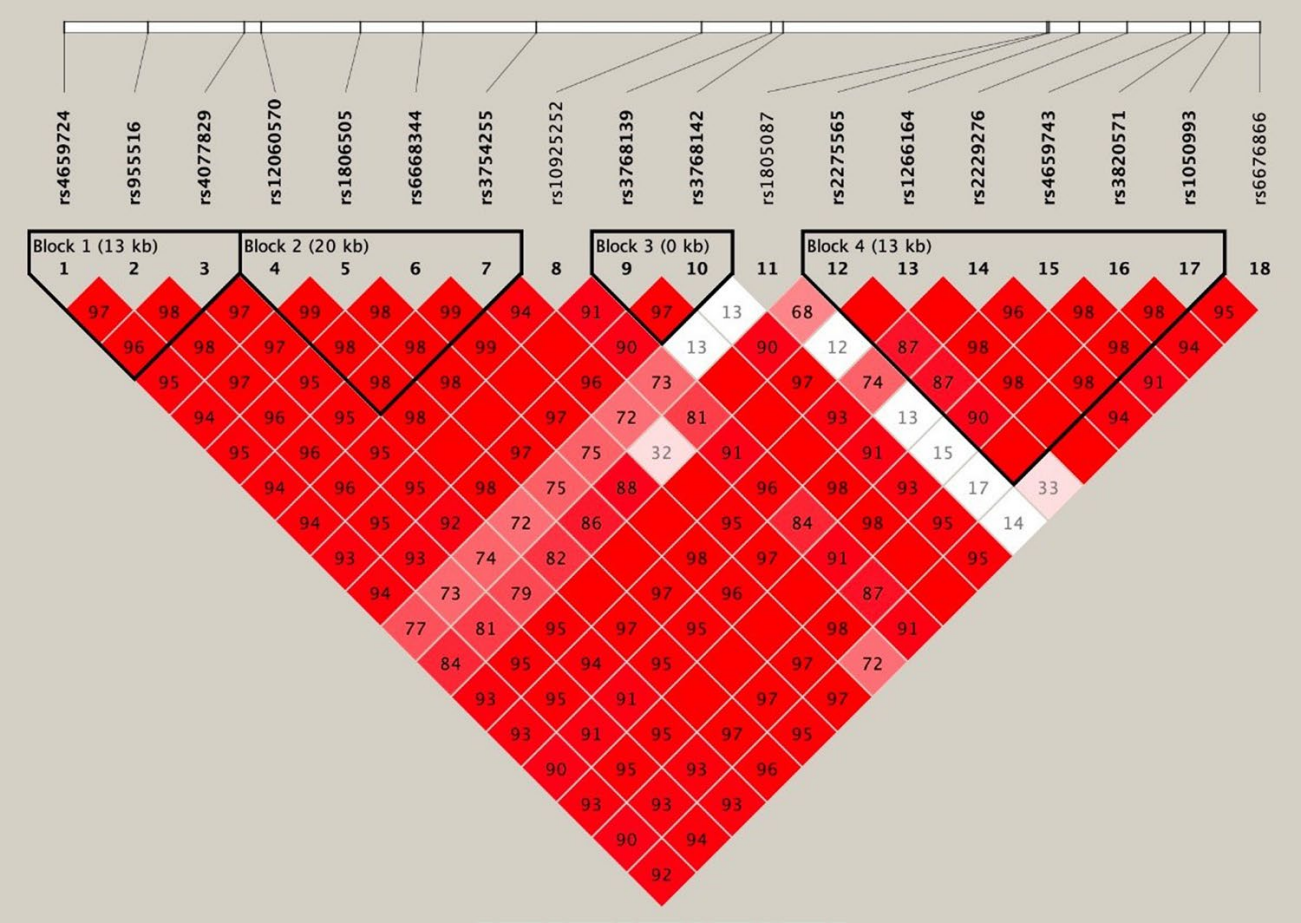
Linkage disequilibrium (LD) analysis of the MTHFR SNPs in both populations.

As shown in Fig. 1, these SNPs constructed four potential linkage disequilibrium blocks. The haplotype frequencies of *MTR* genetic polymorphisms were shown in Table 4. For the risk of CHD, three haplotypes of G-A-T (involving rs4659724, rs95516 and rs4077829; OR = 5.48, 95% CI 2.58–11.66), G-C-A-T-T-G (involving rs2275565, rs1266164, rs2229276, rs4659743, rs3820571 and rs1050993; OR = 0.78, 95% CI 0.63–0.97) and T-C-A-T-T-G (involving rs2275565, rs1266164, rs2229276, rs4659743, rs3820571 and rs1050993; OR = 1.60, 95% CI 1.26–2.04) were identified.

**Table 4:** Haplotype frequencies of *MTR* genetic polymorphisms.

Haplotypes	Case (%)	Control (%)	aOR (95%CI) ^b^	*P*	FDR_*P*^a^
rs4659724-rs95516-rs4077829					
GTG	700.9(56.7%)	689.8(55.8%)	1		
AAT	477.9(38.7%)	521.8(42.2%)	0.90 (0.77–1.06)	0.072	0.144
GAT	45.1(3.7%)	8.1(0.7%)	**5.48(2.58–11.66)**	** < 0.001**	** < 0.001**
rs12060570-rs1806505-rs6668344-rs3754255					
GCCC	588.9(47.6%)	591.9(47.7%)	1		
CTTT	501.9(40.6%)	533.9(43.1%)	0.95(0.80–1.12)	0.217	0.362
GCCT	85.7(6.9%)	60.0(4.8%)	**1.44(1.01–2.03)**	**0.027**	0.068
GCTT	51.4(4.2%)	48.1(3.9%)	1.07(0.71–1.62)	0.721	0.801
rs3768139-rs3768142					
CT	768.3(62.0%)	782.4(63.1%)	1		
CG	238.7(19.3%)	236.6(19.1%)	1.03(0.84–1.26)	0.916	0.916
GG	232.3(18.7%)	214.4(17.3%)	1.10(0.89–1.36)	0.349	0.499
rs2275565-rs1266164-rs2229276-rs4659743-rs3820571-rs1050993					
GCGTTG	554.9(45.0%)	579.3(46.9%)	1		
GCATTG	212.1(17.2%)	282.7(22.9%)	**0.78(0.63–0.97)**	** < 0.001**	** < 0.001**
GTAAGA	228.0(18.5%)	213.0(17.2%)	1.12(0.90–1.39)	0.420	0.525
TCATTG	213.9(17.3%)	139.3(11.3%)	**1.60(1.26–2.04)**	** < 0.001**	** < 0.001**

aOR ,adjusted odds ratio; CI, confidence interval.

^a^ *P* < 0.05 was considered to indicate a statistically significant difference;

^b^ The aOR values and 95% CIs were calculated using binary logistic regression and adjusted for residence, BMI before pregnancy, history of gestational diabetes mellitus, history of gestational hypertension, history of consanguineous marriage, family history of congenital malformations, cold before pregnancy, smoking before pregnancy, alcohol drinking before pregnancy, and folate supplement before or during pregnancy.

Significant values are in [bold].

## Discussion

This study explored the association between 18 SNPs of *MTR* gene with the risk of CHD. After adjustment for potential confounders, we found a small number of significant positive associations among our SNP analyses which seemed consistent with hypothesis that mutant genotypes increased the risk of CHD. Besides, the haplotype analysis showed that G-A-T (OR = 5.48, 95% CI 2.58–11.66), G-C-A-T-T-G (OR = 0.78, 95% CI 0.63–0.97) and T-C-A-T-T-G (OR = 1.60, 95% CI 1.26–2.04) were significantly associated with risk of CHD.

Convincing evidence implied that folate deficiency and HHcy were associated with an increased risk of CHD^19^. Accordingly, genes related to folate/Hcy metabolism were attractive candidates for understanding genetic susceptibility factors for CHD. To data, numerous maternal genes associated with folate/Hcy metabolism pathway had been assessed to the genetic risk factors of CHD, such as the genes of methylene-tetrahydrofolate reductase (*MTHFR*) and cystathionine beta synthase (*CBS*)^20,21^. It was demonstrated that periconceptional folic acid supplementation was associated with a decreased risk of CHD but this association could be modified by variants of the infant MTR gene^22^. Thus, the present study was the first time to investigate the 18 SNPs of *MTR* gene in infant and the risk of CHD. Our study indicated that *MTR* SNPs at rs1805087 and rs2275565 may be associated with the risk of CHD. After controlling for the confounding factors, polymorphisms of *MTR* gene at rs1805087 and rs2275565 trended to increase the risk of CHD in the mutant genotypes (GG vs. AA at rs1805087, aOR = 1.812; TT vs. GG at rs2275565, aOR = 1.679). Published literature had already reported some gene polymorphisms of *MTR* on susceptibility to CHD. They declared that rs28372871 (+ 186 T > G) and rs1131450 (+ 905G > A) variants of *MTR* were significantly associated with the higher risk of CHD and an increase in plasma Hcy concentration^23,24^. In addition, Deng et al.^22^ suggested that mutant alleles of *MTR* gene at rs1770449 and rs1050993 in infant were associated with an increased risk of CHD. Among the SNP mentioned above, only rs1050993 was included in our study and no association between rs1050993 and the risk of CHD was observed. As far as our knowledge, this present study was the first time to find that polymorphisms of *MTR* gene at rs1805087 and rs2275565 were associated with the higher risk of CHD, and it was also the first time that the association between infant *MTR* gene polymorphism and the susceptibility of CHD was comprehensively evaluated. It could help to provide new candidate loci of *MTR* gene when exploring the genetic susceptibility factor of CHD.

The polymorphism rs1805087 (A2756G) in *MTR* gene had become a hot topic in the study of genetic susceptibility factors for birth defects. The A2756G mutation contributed to the substitution of aspartic acid (A allele) to glycine (G allele)^25^. It was located in a protein region interacting with S-adenosyl methionine (SAM) and auxiliary proteins which were significant for the reducing methylation and reactivating the vitamin B_12_ cofactor, which can be oxidatively inactivated in the catalytic process^26^. Thus, mutants might weaken the binding of SAM and/or auxiliary proteins and increased the level of plasma homocysteine^27^. Concerning associations between the polymorphism *MTR* rs1805087 and the plasma homocysteine level was still controversial^28,29^. Previous studies reported the association between rs1805087 and *MTR*. Dr. Shi^30^ and Dr. Galdieri’s results^31^ suggested that maternal *MTR* gene at rs1805087 could not affect the susceptibility to CHD in offspring. With respect to some subtypes of CHD, a case–control study in Chinese population also showed that *MTR* SNPs at rs1805087 was not associated with the occurrence of VSD^32^. Nevertheless, the polymorphism of *MTR* at rs1805087 were identified to be significantly associated with the higher risk of other diseases, such as preeclampsia and breast cancer^33,34^. Our data suggested that infant *MTR* gene at rs1805087 were associated with the higher risk of CHD which was not reported yet. But these observations will need to be replicated in future studies.

Taking the possible interactions between different SNPs into account, we analyzed the haplotypes of *MTR* gene in CHD. Although in single locus analysis, statistically significant associations between genetic polymorphisms at rs1050993 and the risk of CHD were not observed in our research. Interestingly, our research found that the haplotype block formed by rs2275565, rs1266164, rs2229276, rs4659743, rs3820571 and rs1050993 was associated with risk of CHD. A previous study focusing on the relationship of haplotypes of *MTR* genetic polymorphisms with conotruncal heart defects has provided evidence that haplotypes involving rs2275565, rs1266164, rs2229276, rs4659743, rs3820571 and rs1050993 were associated with the increased risk of conotruncal heart defects. On the other hand, associations of haplotypes including the above-mentioned loci with the decreased risk of spina bifida were observed, whose difference may be related to the haplotype frequency^18^. However, given the different subtypes of congenital defects and physiopathologic pathways, further research is still needed to determine the potential mechanism of the protective effect of the haplotypes on the susceptibility of CHD. Previously, only one study^22^, found that haplotype of C-A-A formed by rs1770449, rs1805087 and rs1050993 in *MTR* gene was associated with the occurrence of CHD. Thus, rs1050993 may affect the risk of CHD through gene interaction. However, more researches and elucidations for rs1050993 in the future are needed to figure out its mechanism.

This case–control study had some limitations. Firstly, the data of this study were mainly from the same hospital, and the sample source may be concentrated in a certain type of population, which may affect the representativeness of the sample and lead to selection bias. Secondly, considering the obvious ethnic and regional differences in *MTR* gene polymorphisms, we recruited the participants restricted to the Han Chinese ethnicity. Thirdly, although adjusting for a variety of potential confounding factors, we still cannot completely exclude the involvement of the possibility of residual confounding. Because confounding factors involved in this research did not account for all the risk factors of CHD.

## Conclusions

The present study is the first to exhaustively estimate the association of 18 SNPs of *MTR* gene with the risk of CHD, which suggests that genetic polymorphisms of *MTR* gene at rs1805087 and rs2275565 are significantly associated with CHD. In addition, our study supports a significant association of three haplotypes of G-A-T (involving rs4659724, rs95516 and rs4077829), G-C-A-T-T-G (involving rs2275565, rs1266164, rs2229276, rs4659743, rs3820571 and rs1050993) and T-C-A-T-T-G (involving rs2275565, rs1266164, rs2229276, rs4659743, rs3820571 and rs1050993) with risk of CHD. However, the limitations in this study should be carefully taken into consideration. In the future, more specific studies in different ethnic populations are required to refine and confirm our findings.

## Data Availability

The datasets presented in this study can be found in online repositories. The name of the repository and accession number can be found below: [European Variation Archive (EVA) repository and Project: PRJEB54935, Analyses: ERZ12298562], [http://ftp.ebi.ac.uk/pub/databases/eva/PRJEB54935/].

## Abbreviations

*MTR*: Methionine synthase
CHD: Congenital heart disease
SNPs: Single nucleotide polymorphisms
Hcy: Homocysteine
HHcy: Hyperhomocysteinemia
HWE: The Hardy–Weinberg equilibrium
FDR_*P*: False discovery rate *P* value
OR: Odds ratio
CIs: Confidence intervals
aOR: Adjusted OR
BMI: Body mass index
MTHFR: Methylene-tetrahydrofolate reductase
CBS: Cystathionine beta synthase
ASD: Atrial septal defect
VSD: Ventricular septal defect
AVSD: Atrioventricular septal defect
PDA: Patent ductus arteriosus
APW: Aorto-pulmonary window
TOF: Tetralogy of Fallot
TGA: Complete transposition of great arteries
SAM: S-adenosyl methionine
